# Unusual presentation of alveolar echinococcosis as prostatic and paraprostatic cysts in a dog

**DOI:** 10.1186/1746-6148-9-159

**Published:** 2013-08-12

**Authors:** Caroline A Geigy, Karolin Kühn, Maja Rütten, Judith Howard, Felix Grimm, Carla Rohrer Bley

**Affiliations:** 1 Division of Radiation Oncology, Vetsuisse Faculty, University of Zurich, Zurich, Switzerland; 2 Division of Diagnostic Imaging, Vetsuisse Faculty, University of Zurich, Zurich, Switzerland; 3 Institute of Veterinary Pathology, Vetsuisse Faculty, University of Zurich, Zurich, Switzerland; 4 Clinical Diagnostic Laboratory, Vetsuisse Faculty, University of Bern, Bern, Switzerland; 5 Institute for Parasitology, Vetsuisse Faculty Zurich, University of Zurich, Zurich, Switzerland

**Keywords:** Echinococcus multilocularis, Prostatic cyst, Paraprostatic cysts, Cytology, Radiology, PCR, Canine alveolar echinococcosis, Dog

## Abstract

**Background:**

Alveolar echinococcosis (AE) is caused by the larval stage (metacestode) of *Echinococcus multilocularis*. The domestic dog can act as a definitive host and harbor adult cestodes in its small intestine or become an aberrant intermediate host carrying larval stages that may cause severe lesions in the liver, lungs and other organs with clinical signs similar to AE in humans.

**Case presentation:**

A case of canine AE, affecting the liver and prostate with development of multilocular hydatid paraprostatic cysts and possible lung involvement is described in an 8–year-old neutered male Labrador retriever dog.

The dog presented with progressive weight loss, acute constipation, stranguria and a suspected soft tissue mass in the sublumbar region. Further evaluation included computed tomography of the thorax and abdomen, which revealed cystic changes in the prostate, a paraprostatic cyst, as well as lesions in the liver and lungs. Cytological examination of fine-needle aspirates of the liver, prostate and paraprostatic cyst revealed parasitic hyaline membranes typical of an *Echinococcus* infection and the presence of *E. multilocularis*-DNA was confirmed by PCR.

The dog was treated with albendazole and debulking surgery was considered in case there was a good response to antiparasitic treatment. Constipation and stranguria resolved completely. Six months after the definitive diagnosis, the dog was euthanized due to treatment-resistant ascites and acute anorexia and lethargy.

**Conclusions:**

To the authors’ knowledge, this is the first publication of an *E. multilocularis* infection in a dog causing prostatic and paraprostatic cysts. Although rare, *E. multilocularis* infection should be considered as an extended differential diagnosis in dogs presenting with prostatic and paraprostatic disease, especially in areas where E. multilocularis is endemic.

## Background

Alveolar echinococcosis (AE) is a disease caused by the larval stage (metacestode) of *Echinococcus multilocularis*[1]. The adult tapeworm lives in the small intestine of its definitive host, typically foxes of the genera *Vulpes* and *Alopex*, and various rodents serve as natural intermediate hosts which harbour larval or metacestode stages [2].

Domestic dogs can become definitive hosts by ingesting infected rodents. In Central Europe such as Switzerland, Germany France and the Czech Republic, average prevalence rates of intestinal infection with *E. multilocularis* in dogs are below 0.35%, but may occasionally be higher in certain dog populations with uncontrolled access to rodents [3-8]. After ingestion of *E. multilocularis* eggs from the faeces of an infected definitive host or from the contaminated environment, invasion of parenchymatous organs by metacestode stages occurs in natural as well as in accidental intermediate hosts including humans, monkeys, dogs, pigs and horses [9]. The pathogenic larval stages develop in internal organs, most commonly the liver, leading to severe and sometimes fatal disease if left untreated [1-7,9,10]. In consequence, *E. multilocularis* is a parasite with a significant zoonotic potential and is of clinical relevance in both human and veterinary medicine.

## Case presentation

This case report describes an unusual presentation of canine AE with prostatic and paraprostatic parasitic cysts in an 8-year-old, neutered male Labrador retriever.

The dog was presented for acute constipation, stranguria and a mass in the region of the sublumbar lymph node palpated on rectal examination by the referring veterinarian. In addition, the owner reported weight loss over a period of several months. The dog had been castrated before the age of one year and had no previous history of illness. The dog resided in Switzerland and had a previous travel history to Germany.

On clinical examination, the dog was bright, alert and responsive. Generalised muscle atrophy was evident. Rectal examination revealed an enlarged prostate and a mass in the region of the right sublumbar lymph node. Abdominal palpation revealed cranial organomegaly.

A complete blood count and serum chemistry performed just prior to referral were unremarkable except for a moderate elevation in serum globulins (54 g/L, reference range 25-45 g/L) with a low-normal albumin concentration (22 g/L, reference range 22-39 g/L). Further laboratory analyses included urinalysis and a coagulation profile. The clotting times were within reference ranges. Urine specific gravity was within normal range with 1.026 (1.001-1.065) and dipstick testing revealed a slightly enhanced protein concentration (1+) with unremarkable sediment findings. Urine culture revealed no bacterial growth.

**Figure 1:**
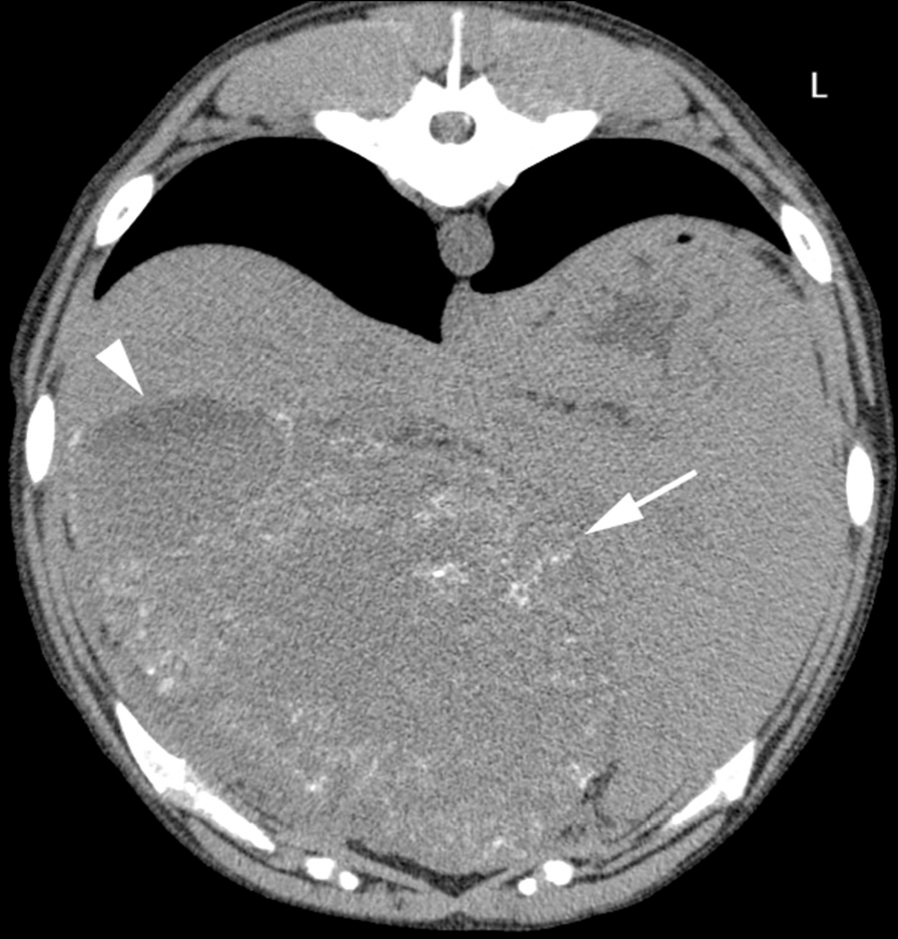
**Transverse CT image of the liver, native study.** Large space-occupying lesions in the liver with hyperattenuating peripheral regions (arrow) and more hypoattenuating central regions (arrowhead). There is a faintly mineral attenuating rim in the periphery of the lesion as well as punctate intralesional mineral attenuating structures.

**Figure 2:**
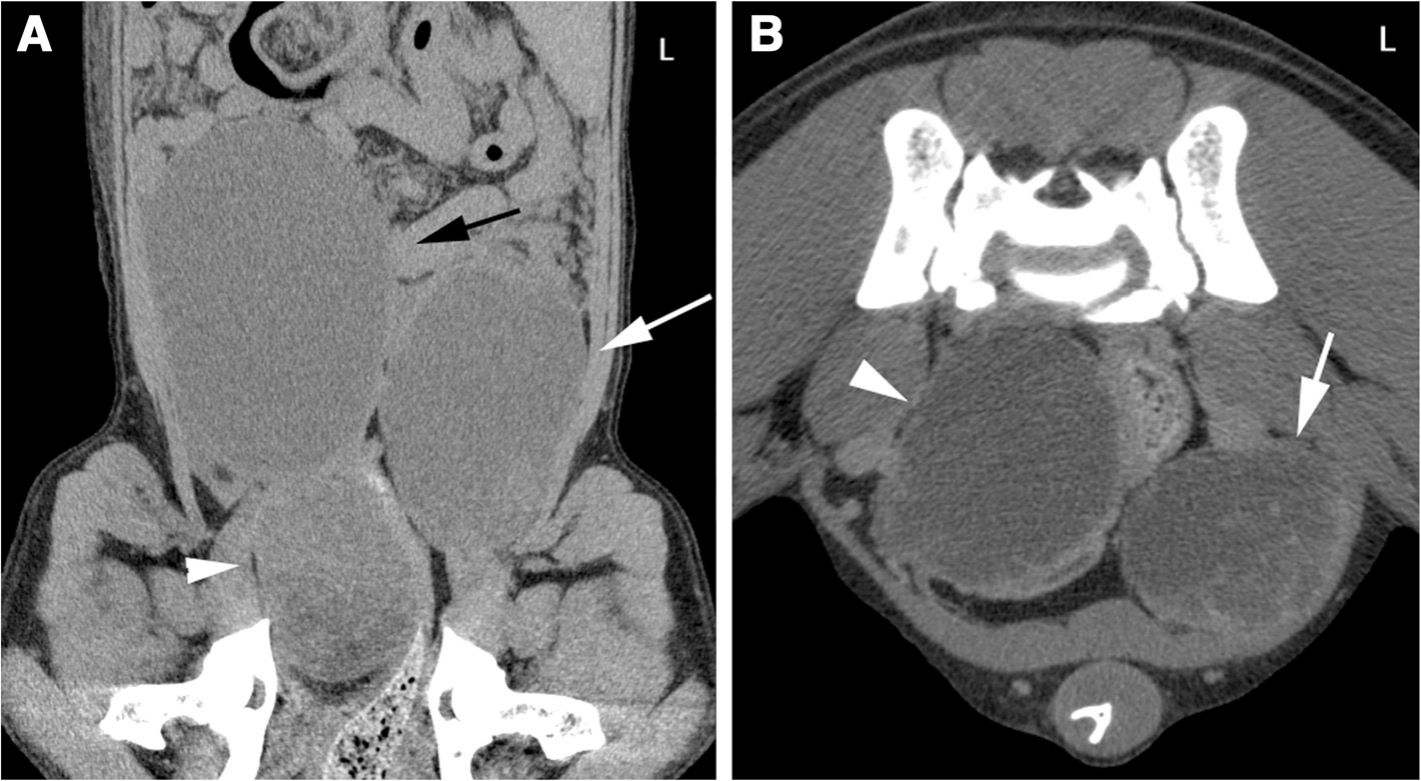
**CT images of the caudal abdomen. A**: Dorsal reconstruction, soft tissue algorithm, post-contrast study. The bladder (black arrow) is located on the right. The prostate (arrowhead) is enlarged with a hypoattenuating centre. Paraprostatic cystic lesion are evident on the left (white arrow). **B**: Transverse image, soft tissue algorithm, post-contrast study. Cystic lesion in the prostate with almost complete replacement of normal parenchyma (arrowhead). Paraprostatic lesion (white arrow) with mild peripheral contrast enhancement.

**Figure 3:**
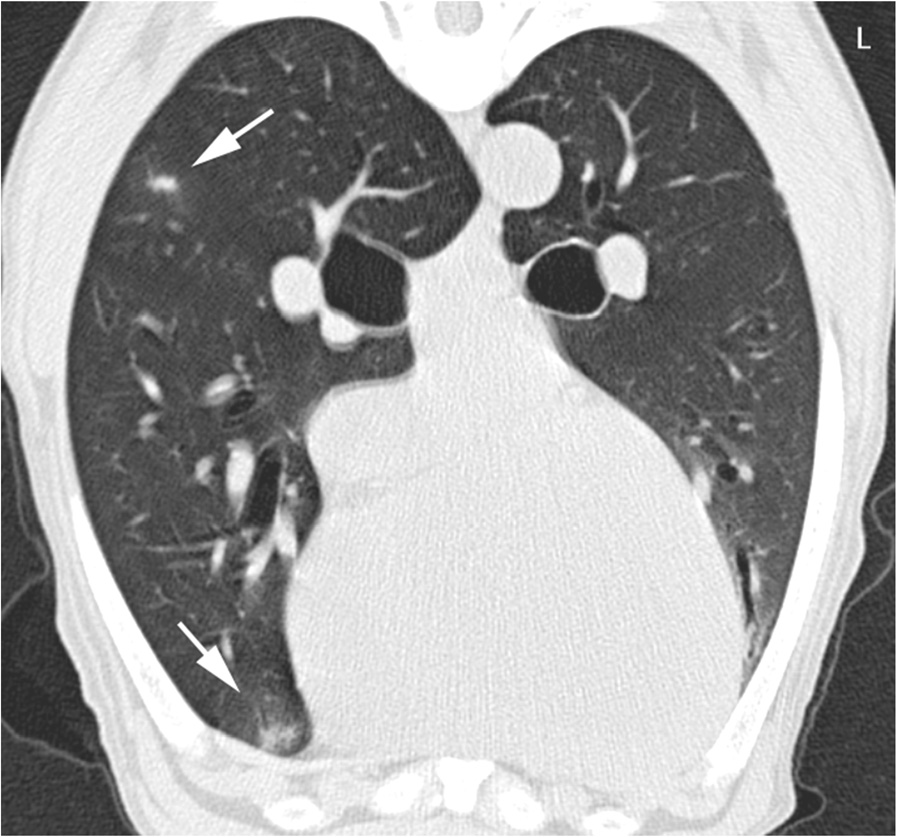
**Transverse CT image of the thorax at the level of the heart, native study.** Poorly defined soft-tissue nodules are evident in the lung (arrows). A mild motion artefact is present.

Abdominal radiographs (left lateral and ventrodorsal projections) performed by the referring veterinarian revealed a large space-occupying lesion in the cranial abdomen, which caused caudal displacement of the stomach and transverse colon. Other findings included moderately decreased abdominal detail and a soft-tissue opacity in the region of the prostate. A CT study (Brilliance CT 16, Philips, Switzerland) of the thorax and abdomen was performed followed by a contrast study (Accupaque ™, 2 ml/kg, 350 mg iodine/ml, GE Healthcare AG, Switzerland) of the abdomen with a delay of 90 seconds post contrast injection. Findings in the abdomen included several large poorly defined cavernous space-occupying lesions in right, central and left ventral aspects of the liver with only little normal liver parenchyma remaining. These showed a hypoattenuating centre (20–40 HU) compared to remaining liver tissue: 50–60 HU, a faintly mineral attenuating rim, punctate intralesional mineral structures and mild peripheral contrast enhancement (Figure 1). The mesentery had a patchy appearance and increased attenuation. The prostate was moderately enlarged and irregular with a large cystic lesion (6.4 cm length × 5 cm height × 4.3 cm width) replacing most of its tissue. In addition, a large paraprostatic cystic lesion (7.8 cm length × 6.1 cm height × 5.4 cm width) (centre 20–50 HU) with a mildly hyperattenuating capsule and mild contrast enhancement, similar to the hepatic lesions, was observed (Figure 2). However, there were no mineralizations within the prostatic or paraprostatic lesions. The urinary bladder was markedly filled and displaced to the right of the cystic lesion, causing a mild curvature to the bladder neck and cranial urethra. The sublumbar lymph nodes appeared unremarkable. The thoracic study revealed several small poorly defined soft-tissue opacities in different lung regions (Figure 3).

Ultrasound-guided fine-needle aspiration was performed on the cystic hepatic, prostatic and paraprostatic lesions.

**Figure 4:**
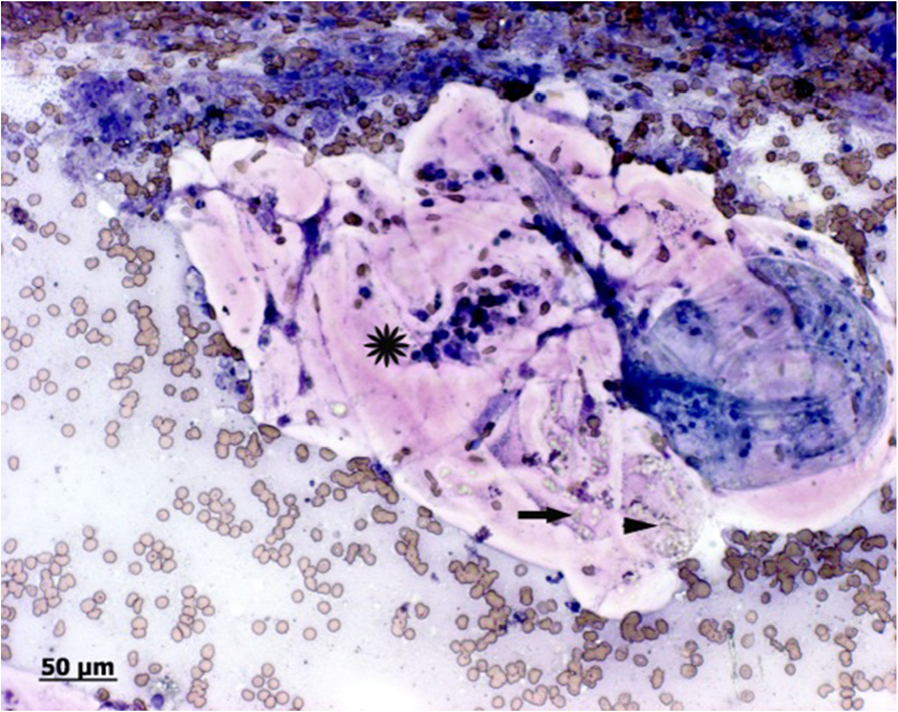
**Photomicrograph of fine needle aspirates of the paraprostatic cyst.** Modified wright stain: Fragments of acidophilic, acellular hyaline membranes (star), folded on itself and containing numerous pale, greenish calcareous corpuscles (arrow). A few greenisch, pale hooklets, consistent with degenerate protoscoleces are present on the right margin (arrowhead). Some erythrocytes (blood contamination) and few neutrophils are visible in the background.

Cytological examination of modified Wright stained smears revealed a moderately modified transudate composed of about 90% nondegenerate neutrophils and approximately 10% macrophages and very few lymphocytes and plasma cells. In addition to nucleated cells, there were multifocally acelluar, acidophilic hyaline membranes, interpreted as fragments of laminated, hyaline membranes of hydatid cysts (Figure 4). The membranes were often highly folded and no intact protoscolices could be seen although some hooklets were observed, suggesting degenerate protoscolices (Figure 4). The membrane-like structures were intensely periodic acid Schiff (PAS) positive. Within these lamellae multiple unstained, crystalline structures of 2-5 μm in diameter were present and interpreted as calcareous corpuscules.

The cytological characteristics and the presence of PAS-positive lamellar structures were highly suggestive for *Echinococcus* species. *E. multilocularis*-DNA was by PCR in all aspirates using primfers H15 and H17 that amplify a E. mulitlocularis specific fragment of the mitochondrial 12SrRNA gene [11].

After draining of the cystic lesions, the dog was able to urinate and defecate without difficulty. Given the extent of the lesions, surgical management was not considered initially feasible. Life-long medical treatment was initiated with oral albendazole (Albex 10%, Chanelle Animal Health Limited, UK) at a dose of 7.5 mg/kg twice daily and the owner was informed that future surgical debulking may become an option if a good response to the antiparasitic treatment was observed. The owner declined a faecal examination and a single dose of 7.5 mg/kg oral praziquantel (Droncit, Bayer (Schweiz) AG, Switzerland) was administered to eliminate any adult stages of *E. multilocularis* that might have been concomitantly present in the dog’s small intestine. In addition, oral enrofloxacin (Xeden, Biokema SA) was prescribed at a dose of 7.5 mg/kg/day for four weeks to treat a possible secondary bacterial infection.

However the dog developed ascites only a few weeks after initiation of albendazole, which was treated by the private practitioner with various diuretics (furosemide, spironolactone and torasemide) over the subsequent few months. The dog was euthanized 6 months after the inital diagnosis due to progressive anorexia and lethargy.

## Discussion

Canine AE causes lesions in the liver, lungs and other organs, similar to the disease observed in humans [12]. In humans the infection is usually subclinical for a long period of time because metacestode growth is commonly slow and the most frequent signs are fatigue and abdominal pain [13]. However clinical AE was found in dogs younger than one year of age, suggesting that the metacestode growth and disease development in the dog progresses more rapidly in dogs than in humans [14-16]. The described dog spent most of his life in Switzerland and travelled to Germany. Intestinal infection with E. multiclocularis in dogs is endemic in both countries [8].

In a retrospective study in 11 dogs, the most frequent clinical finding was progressive abdominal enlargement caused by a hepatic mass [16]. The most frequent laboratory finding was mild to moderate hypoalbuminaemia with concurrent hyperproteinemia, as was the case in the dog described herein [16]. The most common imaging findings were radiographic soft tissue masses in the cranial abdomen that are cavitary on ultrasonographic examination [16]. These were found to contain mineralisations in 5 of 10 dogs on radiographs and 5 of 11 dogs on ultrasound [16]. Small mineralisations of the cavitary lesions and hypoattenuating liquid content are typical radiographic features in CT for AE in children [17] and the ability to detect microcalcifications is considered a major advantage of CT in the diagnosis in human alveolar echinococcosis [18,19]. Similar imaging findings were observed in the case presented herein.

Cytological evaluation of fine-needle aspirates (preferably performed under ultrasound guidance) has been described to be a useful diagnostic tool in canine AE [15]. However, demonstration of *E. multilocularis*-DNA by PCR in aspirated fluids or in tissue biopsies is the preferred method for the definitive identification of *Echinococcus* at the species level in intermediate animal hosts [11,20].

Echinococcosis of the urogenital tract is a rare condition in humans and the kidney is the most frequently affected organ [21-23]. Rare cases of *E. granulosus* (cystic hydatidosis) infections involving the prostate have been described in humans [21,24-28]. However, to the authors’ knowledge prostatic involvement of *E. multilocularis* has not been described in humans or dogs.

Prostatic cysts in dogs are divided in multiple cysts associated with prostatic hyperplasia, retention cysts, paraprostatic cysts and cysts associated with squamous metaplasia [29]. However the aetiology of paraprostatic cysts is poorly understood. Most cases have been described in older, large breed intact male dogs [30-32] that typically showed signs of tenesmus and dysuria due to encroachment of the colon and urethra by the cystic masses [31-33], as was seen in the dog presented in this report. It has been suggested that such cysts arise from remnants of paramesonephric duct. Cystic enlargements in older dogs are reported to be stimulated by hyperoestrogenism associated with Sertoli cell tumors [34]. Although only 2 cases of paraprostatic cysts have been described in neutered male dogs [32], they should be considered a differential diagnosis in castrated male dogs with large prostatic cystic lesions [31,32,34,35]. Moreover, normal paraprostatic cysts may also be mineralized or even show bone formation. This finding cannot therefore be considered an indicator of a parasite lesion. Other large cavitary or cystic lesions of the prostate include abscess and neoplasia or cystic uterus masculinus.

## Conclusions

To the authors’ knowledge, this is the first published case describing an *E. multilocularis* infection associated with prostatic and paraprostatic multilocular cysts. Although a rare manifestation, this parasitic disease should be included as a possible differential diagnosis in dogs with prostatic or paraprostatic cysts that have resided in endemic areas.

## Competing interests

The authors declared that they have no competing interest.

## Authors’ contributions

CG: responsible veterinarian for the patient, writer of the manuscript, submission of the article. KK: Performed the CT on the dog, responsible for the radiologic diagnosis and content of the document, responsible for the radiologic in-put, performed cytology of the cystic lesions, substantive input to the publication (radiology). MR: Responsible for the cytologic diagnosis and content of the document, wrote all details about cytology, substantive input to the publication (clinical pathology). JH: Has been involved in drafting the manuscript an revising it critically for important intellectual content, native English speaker, substantive input to the publication. FG: His laboratory performed the PCR on the cytology smear, substantive input to the publication (parasitologic aspect). CR: Has been involved in drafting the manuscript an revising it critically for important intellectual content. All authors have read and approved the final manuscript.
